# Assessing the mechanism of osteosarcoma induced by long-term PET exposure: prediction from combined network toxicology, machine learning and molecular docking

**DOI:** 10.1016/j.jbo.2025.100714

**Published:** 2025-09-22

**Authors:** Yu Qiao, Fahu Yuan, Anna Curto-Vilalta, Rüdiger von Eisenhart-Rothe, Florian Hinterwimmer

**Affiliations:** a Department of Orthopaedics and Sports Orthopaedics, TUM University Hospital, School of Medicine and Health, Technical University of Munich, Munich, Germany; b School of Medicine, Jianghan University, Wuhan, China; c Institute for AI and Informatics in Medicine, TUM University Hospital, School of Medicine and Health, Technical University of Munich, Munich, Germany

**Keywords:** Polyethylene terephthalate, Osteosarcoma, Network toxicology, Machine learning, Molecular docking

## Abstract

**Objective:**

Polyethylene terephthalate (PET) has emerged as a focal point in addressing global pollution and a critical environmental issue due to its potential health hazards. However, its role in the pathogenesis of osteosarcoma (OS) and the underlying molecular mechanisms remain largely unexplored, further highlighting the necessity of assessing its molecular toxicity.

**Methods:**

This study integrated network toxicology, machine learning, molecular docking, and CIBERSORT-based immune infiltration analysis to systematically investigate the potential impact of PET exposure on contracting OS, elucidating its biological functions, signaling mechanisms, and immune microenvironment. Molecular docking was further applied to characterize the binding properties of PET with hub proteins, and potential therapeutic agents for OS were predicted.

**Results:**

We identified 12 potential key targets of OS associated with PET exposure and, through machine learning models, selected six hub genes (i.e., BCAT1, CDK4, CSF1R, CXCR4, MYB, and PRTN3). Gene Ontology (GO) and kyoto encyclopedia of genes and genomes (KEGG) analyses were conducted to elucidate the roles of these genes in biological processes, cellular components, molecular functions, and signaling pathways. Molecular docking results revealed that PET exhibits high specificity in binding to these hub genes, particularly by interacting with CSF1R (−8.312 kcal/mol), potentially activating the PI3K-Akt signaling pathway and modulating the OS immune microenvironment to promote tumor progression through multiple mechanisms. Furthermore, drug prediction analysis identified *p*-Benzoquinone and JNK-9L as potential therapeutic candidates for OS.

**Conclusion:**

This study reveals that PET may play a critical role in OS development by regulating hub genes and signaling pathways. Molecular docking analysis demonstrates that PET can tightly bind to specific target proteins, suggesting a potential molecular mechanism underlying OS progression. These findings provide a scientific basis for further evaluating PET-related health risks and offer theoretical support for the development of future prevention and treatment strategies.

## Highlights

• Interdisciplinary integration: This study predicts the potential effects of long-term exposure to polyethylene terephthalate (PET) on osteosarcoma (OS).
• Discovery of regulatory mechanisms: This research unveils a multifaceted mechanism wherein PET may induces conformational changes in CSF1R, activating the PI3K-Akt signaling pathway and modulating the tumor immune microenvironment.
• Environmental health implications: By analyzing and predicting the potential health risks of PET exposure, this research enhances public awareness of environmental protection.

## Graphical abstract

**Figure:**
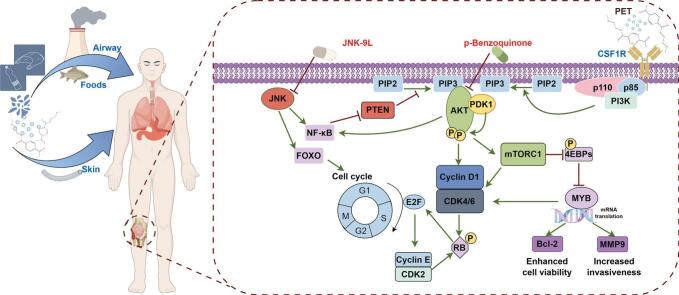

## 1 Introduction

As plastic pollution becomes increasingly severe, regulatory efforts targeting plastic products are being strengthened worldwide. In 2024, the European Union introduced new regulations requiring beverage suppliers to attach bottle caps to their containers to reduce plastic pollution and improve recycling efficiency. This policy highlights the growing global concern over the environmental impact of plastic products. It is estimated that over 350 million tons of plastic are produced worldwide each year, with polyethylene terephthalate (PET) being a major component [1]. Due to its excellent mechanical strength, chemical stability, and high transparency, PET is widely used in food and beverage packaging, textile fibers, personal care products, medical devices, and toys [2]. However, PET degradation may release harmful substances such as antimony (Sb), acetaldehyde, and microplastics, and long-term exposure to these substances may affect human health and even increase the risk of certain disease [3,4].

In recent years, the widespread distribution of plastic pollutants in the environment and their potential impacts on human health have attracted growing attention from the scientific community. Microplastics are widely recognized as emerging environmental pollutants, usually referring to plastic particles or fibers with a particle size of less than 5 mm [5]. These microplastics can be produced through environmental degradation, plastic aging, or industrial additions, and are commonly found in drinking water, food, air, and even medical supplies [[6], [7], [8]]. Human exposure to microplastics primarily occurs through ingestion, inhalation, and dermal contact, with PET microplastics being among the most frequently encountered types. Notably, exposure risk is heightened in high-temperature environments, where elevated temperatures accelerate the release and migration of microplastic particles and associated harmful small molecules [9]. Numerous studies have detected microplastics in various human tissues, including blood, alveoli, placenta, breast milk, intestinal contents, lymphatic tissues, and bone marrow [10]. These findings suggest that microplastics possess the capacity for widespread systemic distribution and may even cross the blood–brain barrier and placental barrier, enabling maternal-fetal transmission and potentially affecting early fetal development [11,12].

The potential threats of microplastics to human health manifest on multiple levels. First, once microplastics enter human tissues, they may cause mechanical irritation or structural damage to cells, thereby inducing localized inflammation and cellular stress responses [13,14]. Second, due to their high surface area and adsorption capacity, microplastics can carry environmental pollutants such as polycyclic aromatic hydrocarbons, heavy metals, antibiotic residues, and pathogenic microorganisms, enhancing their toxicity and persistence within the body [15]. Additionally, PET microplastics may release toxic small molecules during degradation, such as acetaldehyde and antimony ions, which have been shown to exert cytotoxic, endocrine-disrupting, and even carcinogenic effects [16]. Animal studies have further demonstrated that microplastic exposure can trigger immune activation, increase oxidative stress, disrupt gut microbiota composition, and interfere with lipid metabolism, suggesting their potential involvement in the development of metabolic disorders, immune-related diseases, and cancers [[17], [18], [19]]. Notably, emerging evidence also indicates that microplastics may impair the function of osteoblasts and osteoclasts, hinder bone remodeling, and promote inflammation within bone tissue, thereby disturbing bone metabolic homeostasis and possibly contributing to the progression of osteoporosis, osteitis, or even bone tumors [20,21].

In this context, osteosarcoma (OS), a highly malignant primary bone tumor originating from osteoblasts, has attracted attention regarding its potential association with environmental factors, particularly emerging pollutants. OS predominantly affects children and adolescents, and its pathogenesis is complex, involving interactions among genetic, metabolic, and microenvironmental factors. Although advances in surgery, chemotherapy, radiotherapy, and targeted therapies have improved treatment outcomes in recent years, the overall prognosis remains poor, with high rates of local recurrence and distant metastasis, and unsatisfactory five-year survival rates [22].

There is increasing evidence that environmental exposures, especially heavy metals and organic pollutants, are closely related to the occurrence of bone diseases and tumors. Among these, the effects of plastic-derived substances and their degradation products on the skeletal system have gained growing attention [23,24]. Recent studies suggest that microplastics may disrupt bone homeostasis by inducing systemic inflammation, impairing osteoblast function, or damaging the bone microenvironment through oxidative stress, thereby increasing the risk of bone disorders [25]. Degradation by-products of PET, including antimony, acetaldehyde, and various microplastic particles, have been shown to exert immunotoxic and metabolic toxicity in multiple animal models [26,27]. However, whether long-term exposure to PET can induce the development of OS remains an open question, as no in-depth or systematic studies have yet addressed this issue. This represents an important gap in current scientific understanding.

**Fig. 1:**
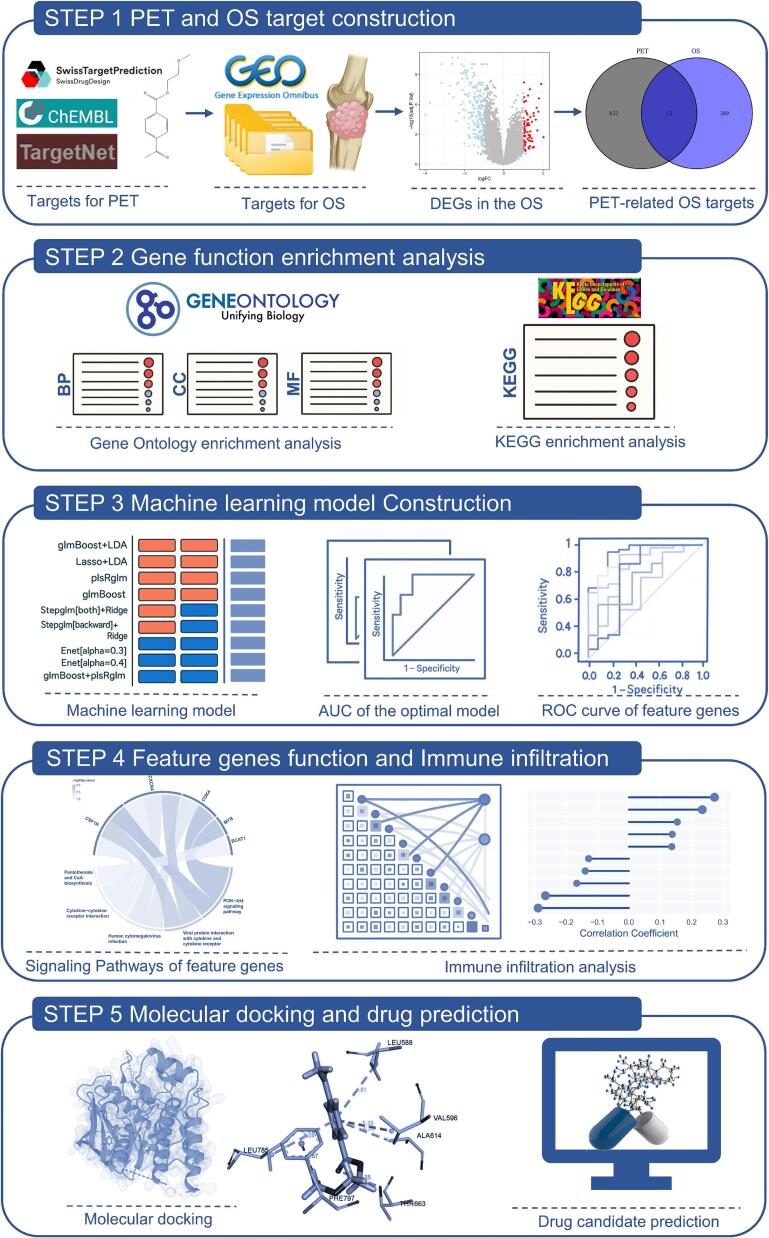
Overview of the methodological framework used to investigate the potential toxicological mechanisms of polyethylene terephthalate (PET) exposure in osteosarcoma (OS). AUC: Area Under the Curve; DEGs: Differentially expressed genes; ROC: Receiver Operating Characteristic; KEGG: Kyoto Encyclopedia of Genes and Genomes.

Recent advances in network toxicology and computational biology have revolutionized the study of environmental toxins and their interactions with biological systems [28]. By fitting models onto transcriptomic data, using machine learning algorithms and molecular docking simulations, researchers are able to predict potential toxicological pathways and identify key molecular targets with high accuracy [29,30]. These approaches have shown significant value in elucidating the mechanisms of chemical-induced carcinogenesis and immune dysregulation. In this study, we identified PET-related molecular targets and their interactions with OS-related genes through bioinformatics, predicted and validated PET-induced OS hub genes using advanced machine learning models, and finally characterized the binding interactions between PET and hub gene-encoded proteins through molecular docking simulations. This is our first attempt to bridge the gap between environmental toxicology and OS biology, providing new insights to assess the potential mechanisms of long-term PET exposure-induced OS. Meanwhile, our findings also provide a theoretical basis for public health policy and prevention of OS, and open up new avenues for safety assessment of PET and environmentally healthy disposal of PET. The workflow of this study is shown in Fig. 1.

## 2 Methods

Based on the preliminary recognition that PET exposure may contribute to the development of OS, we systematically analyzed the potential toxicity of PET in conjunction with gene expression profiling of OS. This study utilized transcriptomic data integrated with machine learning algorithms, immune cell infiltration analysis, and drug repurposing prediction to explore the mechanisms through which PET may contribute to OS development.

### 2.1 Data collection

To investigate the potential mechanisms by which PET exposure causes OS, we integrated datasets from several public databases and datasets. Detailed information on all databases and datasets, including the microarray platform, information is given in Table 1.

**Table 1:** Summary of databases and GEO datasets included in this study.

Name of database	Dataset	Source/platform	Purpose/application
PubChem	Databases	https://pubchem.ncbi.nlm.nih.gov/	Obtaining the molecular structure and SMILES of PET
TagetNet	Databases	https://targetnet.scbdd.com.	Predicting PET target proteins
CheMBL	Databases	https://www.ebi.ac.uk/chembl/	Predicting PET target proteins
SwissTargetPrediction	Databases	https://www.swisstargetprediction.ch	Predicting PET target proteins
RCSB PDB	Databases	https://www.rcsb.org/	Obtaining the crystal structure of hub target proteins
DSigDB	Databases	https://dsigdb.tanlab.org/DSigDBv1.0/	Obtaining genetic data relevant to drug prediction
GEO^1^	GSE19276	GPL6848	Obtaining key genes in OS; Training set
GEO^1^	GSE36001	GPL6102	Obtaining key genes in OS; Training set
GEO^1^	GSE33383	GPL6801	Test set

^1^GEO = Gene expression omnibus.

### 2.2 Acquisition of PET targets

The molecular structure of PET and Simplified Molecular Input Line Entry System (SMILES) “CC(=O)C1=CC=C(C=C1)C(=O)OCCOC” were obtained from the PubChem database (3D and 2D molecular structures are provided in Fig.S1) [31]. The TagetNet, CheMBL and SwissTargetPrediction databases were used to obtain potential impact targets for PET [[32], [33], [34]]. To ensure biological relevance, we set the target biocategory to “Homo sapiens”, set the predictive probability >0.6 for targets from the TagetNet database, and converted the IDs of all targets using UniProt and repeated the validation. Finally, the targets from different databases were merged to form a comprehensive PET target database after removing duplicates.

### 2.3 Acquisition of OS-related targets

To identify differentially expressed genes (DEGs) associated with OS, three OS datasets (GSE19276, GSE36001, and GSE33383) were retrieved from the gene expression omnibus (GEO) database. To obtain the key genes associated with PET in OS using machine learning methods, we used GSE19276 and GSE36001 as the training group and GSE33383 as the test group. The training set contained 63 OS and 11 control samples, while the test set contained 15 control and 84 OS samples. To ensure data consistency, we corrected for batch effects in the training dataset using the SVA package [35]. Additionally, the ComBat algorithm was applied to further adjust batch effects, enhancing comparability across samples. The distribution of corrected expression data was visualized using boxplots and principal component analysis (PCA).

### 2.4 Acquisition of OS targets related to PET

Differential expression analysis of the OS training dataset was performed using the limma package [36]. DEGs were identified using the Benjamin-Hochberg method with the adjusted *p*-value < 0.05 and |logFC| > 1 as the selection threshold, and the ggplot2 and pheatmap packages were used to visualize the DEGs of OS [36]. To identify PET-related targets associated with OS, the VennDiagram package was used to compare OS-related DEGs with PET targets and thus explore the potential mechanisms of PET-induced OS toxicity [37].

### 2.5 GO and KEGG pathway enrichment analysis

To elucidate the biological functions and signaling pathways of the potential target genes, Gene Ontology (GO) and Kyoto Encyclopedia of Genes and Genomes (KEGG) enrichment analyses were performed with the clusterProfiler package, applying a threshold of *p* < 0.05 for statistical significance [[38], [39], [40]]. GO analysis was used to predict the functional categories of the genes, including biological processes (BP), cellular components (CC), and molecular functions (MF). KEGG analysis was used to identify key signaling pathways associated with both OS and PET exposure. The top five enriched biological functions from GO analysis and the top 20 KEGG pathways were visualized for further interpretation.

### 2.6 Optimal machine learning model construction and hub genes screening

To identify the optimal OS potential genes associated with PET exposure, we constructed 113 predictive models based on the OS training dataset. Specifically, we employed the twelve machine learning algorithms, Lasso, SVM, RF, glmBoost, Stepglm, Ridge, Enet, plsRglm, GBM, LDA, XGBoost, and Naive Bayes, to analyze feature genes in both the training and test sets [41]. All machine learning model configurations, including algorithm types, packages, and parameter settings, are summarized in Supplementary Table S1. The model with the highest area under the curve (AUC) was selected for evaluation in both the training and an external test set. Receiver operating characteristic (ROC) curves were generated for each dataset to assess model classification performance, and AUC values were calculated to quantify the model’s ability to distinguish OS and control samples. This process enabled the identification of the most robust feature gene-related model, followed by further assessment of its predictive performance. Additionally, a confusion matrix was generated to evaluate model accuracy by comparing predicted class labels with actual labels in both the training and test sets.

### 2.7 Analysis of hub genes function and the immune microenvironment

The clusterProfiler package was used to perform KEGG enrichment analysis on hub genes, while the cell-type identification by estimating relative subsets of RNA transcripts (CIBERSORT) algorithm was employed to assess immune cell infiltration in control and OS tissues [38,42]. To minimize technical variations between different datasets, quantile normalization and batch effect correction were applied using the ComBat algorithm [43]. To ensure result robustness and reproducibility, the CIBERSORT algorithm was run with 1,000 permutations. The analysis output included *p*-values, correlation coefficients, and RMSE values, with only samples meeting the significance threshold (*p*<0.05) considered for further analysis. Additionally, we investigated the correlation between hub genes (BCAT1, CDK4, CSF1R, CXCR4, MYB, and PRTN3) and various immune cell types. This integrated approach provides new insights into the complex interactions between hub genes and immune cell infiltration in OS, potentially identifying novel therapeutic targets for modulating the tumor immune microenvironment.

### 2.8 Molecular docking validation of PET with hub genes

To evaluate the impact of PET on hub genes, we used Autodock Vina 1.2.2, a computational protein–ligand docking software [44]. First, we retrieved the crystal structure PDB files of hub target proteins from the RCSB PDB database, and the ligand files in SDF format of the PET 3D structures from the PubChem database [45]. The protein and ligand files were prepared by converting all protein and molecular files into PDBQT format. Water molecules were removed, and polar hydrogen atoms were added. The grid box was centered to cover the protein’s structural domain, allowing for flexible molecular movement. A cubic docking pocket of 30 Å × 30 Å × 30 Å was defined, with a grid spacing of 0.05 nm. Finally, the docking results were visualized to assess the binding interactions between PET and the hub genes.

### 2.9 Drug prediction

The 688,780 drug prediction-related gene data obtained from the drug signatures database (DSigDB) were compared with hub genes [46]. Setting a corrected *p*-value < 0.01 was considered statistically significant and ranked to visualize the top 10 predicted drugs.

## 3 Results

### 3.1 Identification of OS-related targets induced by PET exposure

**Fig. 2:**
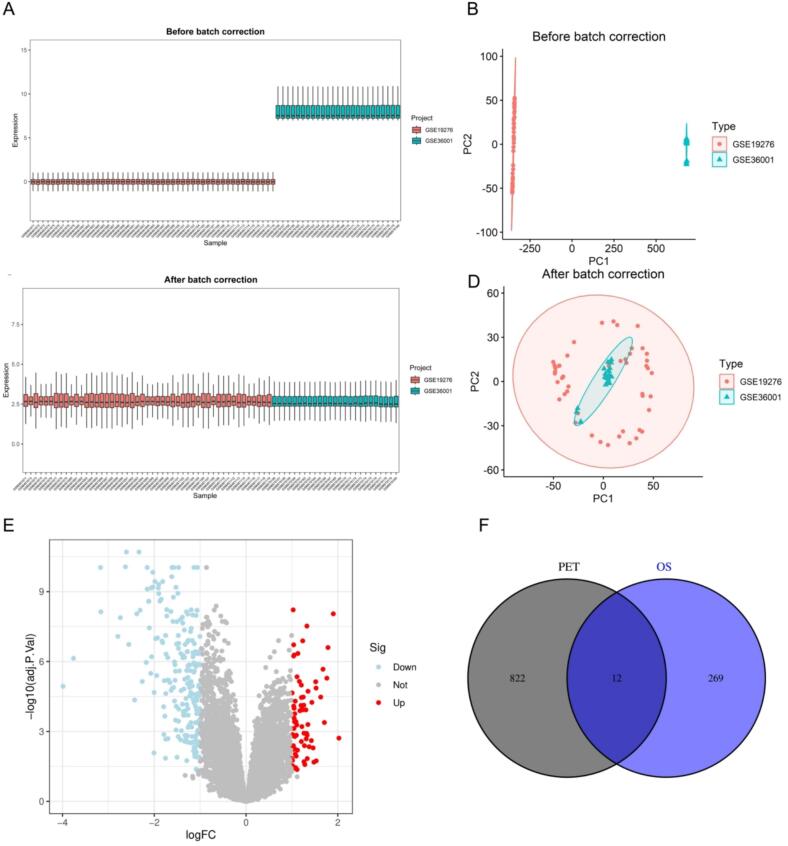
Acquisition of polyethylene terephthalate (PET) exposure-related osteosarcoma (OS) targets. (A, C) Boxplots displaying the distribution of expression values before (A) and after (C) normalization in the training dataset, showing improved consistency and comparability across samples. (B, D) Principal component analysis plots before (B) and after (D) normalization, indicating enhanced clustering of OS and control samples post-processing. (E) Volcano plot presenting the DEGs between OS and normal samples. Red dots represent upregulated genes, green dots represent downregulated genes, and grey dots represent non-significant genes (F) The Venn diagram shows 12 intersecting genes between the predicted PET-related toxic targets and OS-related DEGs. (For interpretation of the references to colour in this figure legend, the reader is referred to the web version of this article.)

In this study, we screened 834 potential targets associated with PET using the TargetNet, ChEMBL, and SwissTargetPrediction databases. These targets may mediate potential molecular interactions between PET and human proteins. To further explore the relationship between PET exposure and OS, we integrated genomics data from human OS samples extracted from the GEO datasets GSE19276 and GSE36001. This integration aimed to identify OS-related genes. Box plots (Fig. 2A and C) illustrate the distribution of sample expression before and after data integration, while PCA analysis (Fig. 2B and D) demonstrates a significant reduction in batch effects, enhancing sample uniformity.

To further validate the genomics differences between control and OS samples, we conducted differential expression analysis on the integrated dataset, identifying a total of 281 DEGs. A volcano plot was generated to visualize the distribution of these DEGs in OS, with significantly upregulated genes highlighted in red and downregulated genes in blue (Fig. 2E). Additionally, a heatmap was created to display the top 30 differentially expressed genes (Fig.S2). Finally, to establish a potential molecular link between PET exposure and OS, we performed a Venn diagram analysis, comparing the 834 PET-related targets with the 281 OS-associated DEGs. This analysis identified 12 intersection target genes (Fig. 2F). These genes may represent key targets in the pathogenesis of OS with long-term PET exposure.

### 3.2 Enrichment analysis of intersecting target genes

**Fig. 3:**
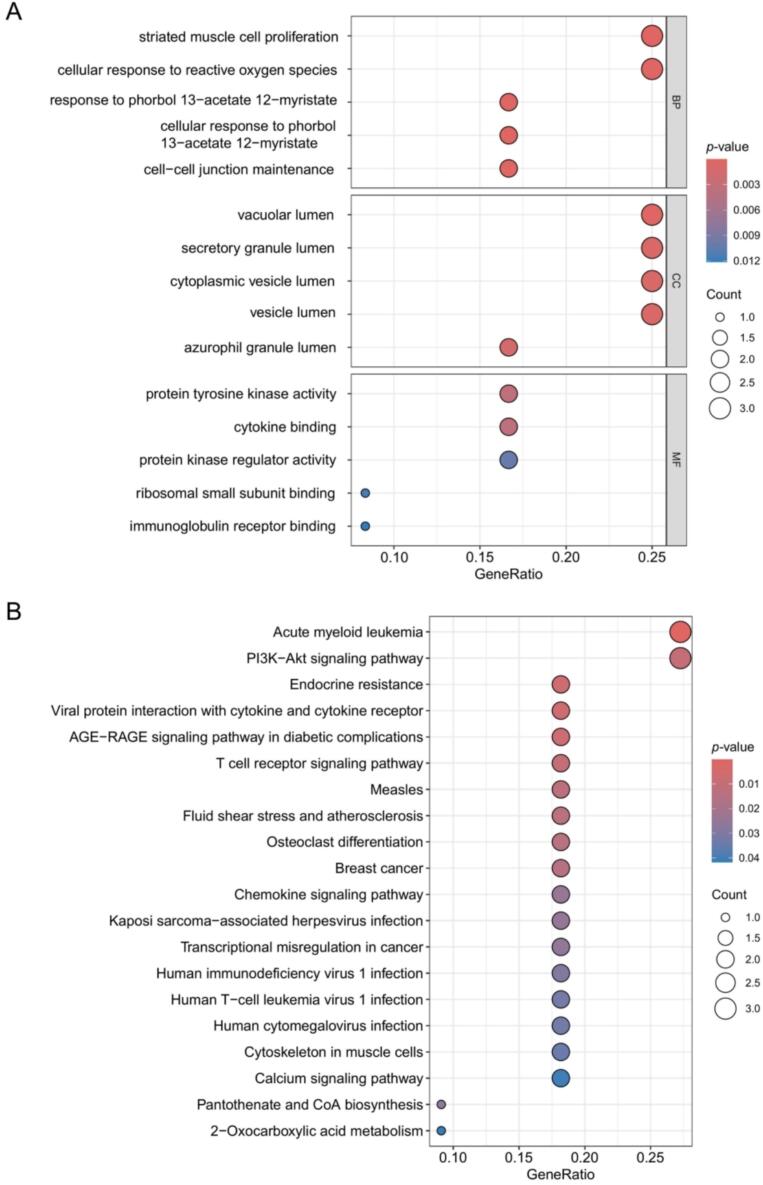
Functional enrichment analysis of genes associated with PET-related OS. (A) Bubble plots presenting the top five enriched terms in each GO category: biological process (BP), cellular component (CC), and molecular function (MF). (B) KEGG pathway analysis highlighting the top 20 significantly enriched signaling pathways. In each bubble plot, the size of the circle represents the number of genes involved in the corresponding term, and the color gradient from blue to red indicates the statistical significance. (For interpretation of the references to colour in this figure legend, the reader is referred to the web version of this article.)

To investigate the biological functions and signaling pathways associated with the 12 potential OS target genes linked to PET exposure, we conducted GO and KEGG enrichment analyses (Fig. 3A, B and Fig. S3). GO analysis indicated that these intersection genes may play a role in the occurrence and development of OS through several biological processes, including cellular response to reactive oxygen species (ROS) [47], protein tyrosine kinase activity [48], cytokine binding [49], protein kinase regulator activity [50], and striated muscle cell proliferation [51]. KEGG analysis further revealed key OS-related signaling pathways, such as the PI3K-Akt signaling pathway [52], endocrine resistance signaling pathway [53], T cell receptor signaling pathway [54], osteoclast differentiation signaling pathway [55], chemokine signaling pathway [56], and transcriptional misregulation in cancer signaling pathway [57]. These findings suggest a strong link between long-term PET exposure and OS development, potentially mediated through these biological functions and pathways.

### 3.3 Machine learning model construction and identification of hub genes

To optimize feature selection of the intersection genes and enhance the predictive accuracy of the hub genes associated with OS due to PET exposure, we implemented a comprehensive machine learning framework. The 113 prediction models were constructed using training and external test datasets derived from the 12 intersection genes. The model with the highest AUC was selected for further evaluation.

**Fig. 4:**
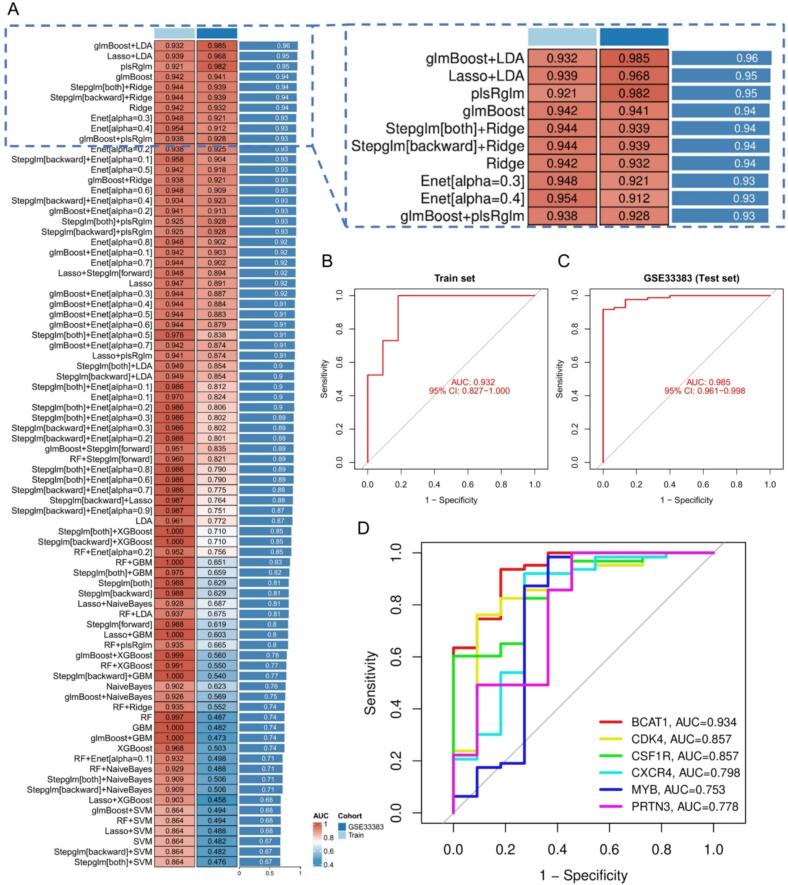
Machine learning-based validation of hub genes. (A) Heatmap showing area under the curve (AUC) values for 113 different machine learning models trained on intersecting genes. Among them, the glmBoost + LDA model has the best performance (AUC = 0.96). (B-C) Receiver Operating Characteristic (ROC) curves comparing model performance on training and testing datasets, respectively. (D) ROC curves assessing the diagnostic value of the hub genes selected according to the glmBoost + LDA model.

Fig. 4A shows a heatmap comparing the AUC values of different models and cohorts. Among them, the glmBoost + LDA model demonstrated the best performance, achieving the highest AUC across all datasets. This model attained an AUC of 0.932 (95 % CI: 0.827–1.000) in the training set, exhibiting strong discriminative power between OS and control samples (Fig. 4B). Additionally, its performance was further validated using an external dataset, where it achieved an AUC of 0.985 (95 % CI: 0.961–0.998) in the GSE33383 dataset (Fig. 4C).

To further evaluate the classification accuracy of the model, we generated confusion matrices for the training set and test cohort (Fig.S4). In the training set, the model correctly classified 7 control samples and 63 OS samples, of which 4 control samples were incorrectly classified as OS samples, resulting in an accuracy of 94.6 %, a sensitivity of 100 %, a specificity of 63.6 %, and an AUC of 0.932 for the training set. In the GSE33383 cohort, the model also showed strong classification accuracy, correctly identifying 79 OS samples, of which 5 OS samples were misclassified as controls and 15 control samples were misclassified as OS samples, resulting in an accuracy of 79.8 %, a sensitivity of 94.1 %, a specificity of 0, and an AUC of 0.985 for the training set.

This strong performance, including the slightly higher AUC observed on the test dataset, can be explained by the interplay between regularization, sample size, and class distribution. The glmBOOST algorithm incorporates intrinsic shrinkage and feature selection mechanisms, which are particularly well-suited for high-dimensional, low-sample-size data such as gene expression profiles [58,59]. This helps prevent overfitting while maintaining discriminative capacity, as confirmed by the training set results in Fig. S4. Moreover, the external test dataset (GSE33383) includes more samples than the training set and preserves a similar case-control ratio, which contributes to more stable and potentially enhanced performance metrics. In particular, the high AUC in the test set reflects the model’s strong ability to correctly identify OS cases, which form the majority class, while the small number of controls (n = 15) increases metric sensitivity. These factors together support the robustness and generalizability of the selected glmBOOST + LDA model.

Based on these results, we selected the glmBoost + LDA model as the optimal model. Using this model, we identified six hub genes: BCAT1, CDK4, CSF1R, CXCR4, MYB, and PRTN3. Fig. 4D presents the ROC curves of these six genes, demonstrating their high diagnostic accuracy. The correlation plot in Fig. S5 further investigates the relationships among these hub genes. The correlation coefficient (r) ranges from –1 to 1, with values closer to 1 or –1 indicating stronger positive or negative linear relationships, respectively. The analysis reveals significant correlations, including a strong positive correlation between CSF1R and PRTN3 (r-value: 0.43, *p* < 0.001), and a strong negative correlation between BCAT1 and PRTN3 (r-value: −0.41, *p* < 0.001).

### 3.4 Analysis of hub genes function and immune microenvironment

**Fig. 5:**
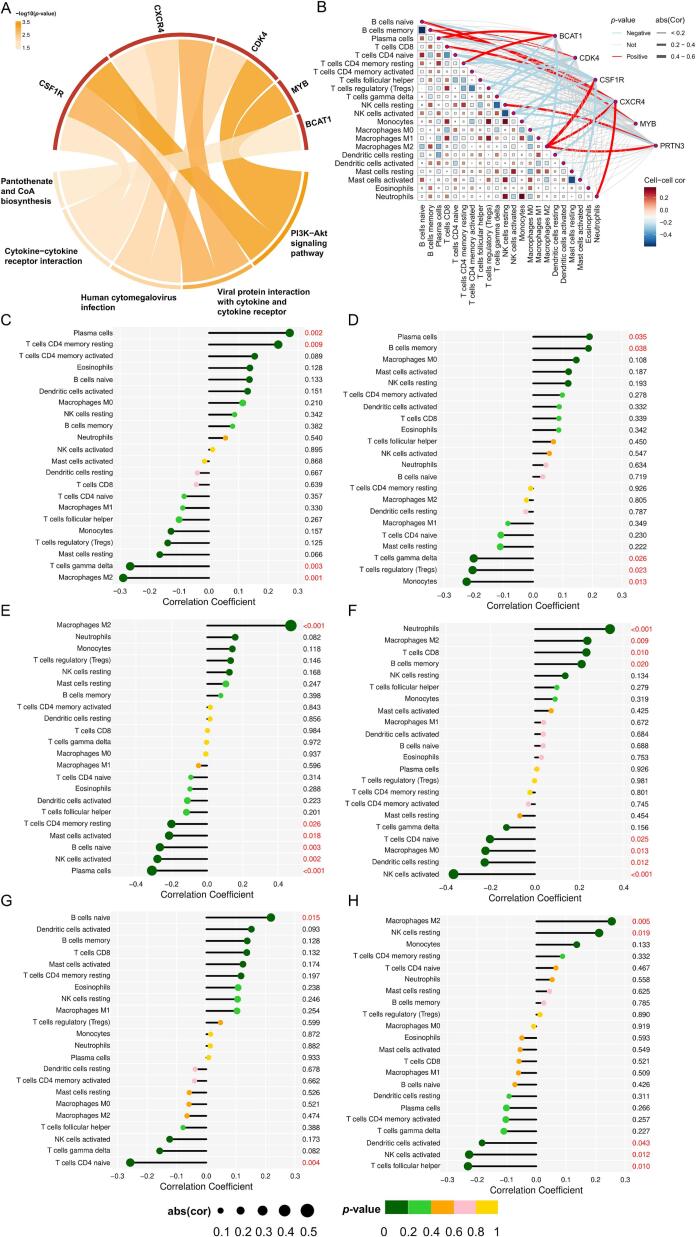
Functional and immune landscape analysis of hub genes. (A) Hub genes KEGG enrichment analysis chord diagram. (B) Reflects the relationship between hub genes and immune cells. Orange lines represent positive associations with immune cells and green lines represent negative associations. Mesh grid colors represent correlation between immune cells, red represents positive correlation and blue represents negative correlation. (C-H) Lollipop plots showing the correlation between the expression levels of BCAT1 (C), CDK4 (D), CSF1R (E), CXCR4 (F), MYB (G), and PRTN3 (H) and the infiltration of 22 immune cells. Circle size indicates the absolute value of the correlation coefficient; circle color indicates the p-value of the correlation test, where *p* < 0.05 (red) indicates a significant association between gene expression and immune cell content. (For interpretation of the references to colour in this figure legend, the reader is referred to the web version of this article.)

We conducted KEGG enrichment analysis on the six potential genes associated with PET exposure-induced OS, revealing a strong correlation with the PI3K-Akt signaling pathway (Fig. 5A). Additionally, we performed immune cell infiltration analysis, which uncovered the potential roles of these hub genes in the OS immune microenvironment and their associations with different immune cell types. Our results indicate that these genes may play a crucial role in regulating immune cell infiltration and shaping the tumor microenvironment (Fig. 5B). Specifically, our findings demonstrated that BCAT1 was positively correlated with Plasma cells and T cells CD4 memory resting, but negatively correlated with Macrophages M2 and T cells gamma delta (Fig. 5C). CDK4 was positively correlated with Plasma cells and B cells memory, but negatively correlated with Monocytes, T cells regulatory (Tregs) and T cells gamma delta (Fig. 5D). CSF1R was positively correlated with Macrophages M2, but negatively correlated with Plasma cells, NK cells activated, B cells naive, Mast cells activated, and T cells CD4 memory resting (Fig. 5E). CXCR4 was positively correlated with Neutrophils, Macrophages M2, T cells CD8 and B cells memory, but negatively correlated with NK cells activated, Dendritic cells memory, Macrophages M0 and T cells CD4 naive (Fig. 5F). MYB positively correlated with B cells naive but negatively correlated with T cells CD4 naive (Fig. 5G). PRTN3 was positively correlated with Macrophages M2 and NK cells resting, but negatively correlated with T cells follicular helper, NK cells activated, and Dendritic cells activated (Fig. 5H). Scatter plots depicting the correlation between hub gene expression and immune cell infiltration are provided in Fig. S6–11. These findings suggest that PET exposure-induced OS may be closely linked to immune cell infiltration, potentially influencing OS occurrence and progression by regulating macrophage polarization, B cell differentiation, T cell activation, and NK cell function.

### 3.5 Molecular docking of PET with hub genes

**Fig. 6:**
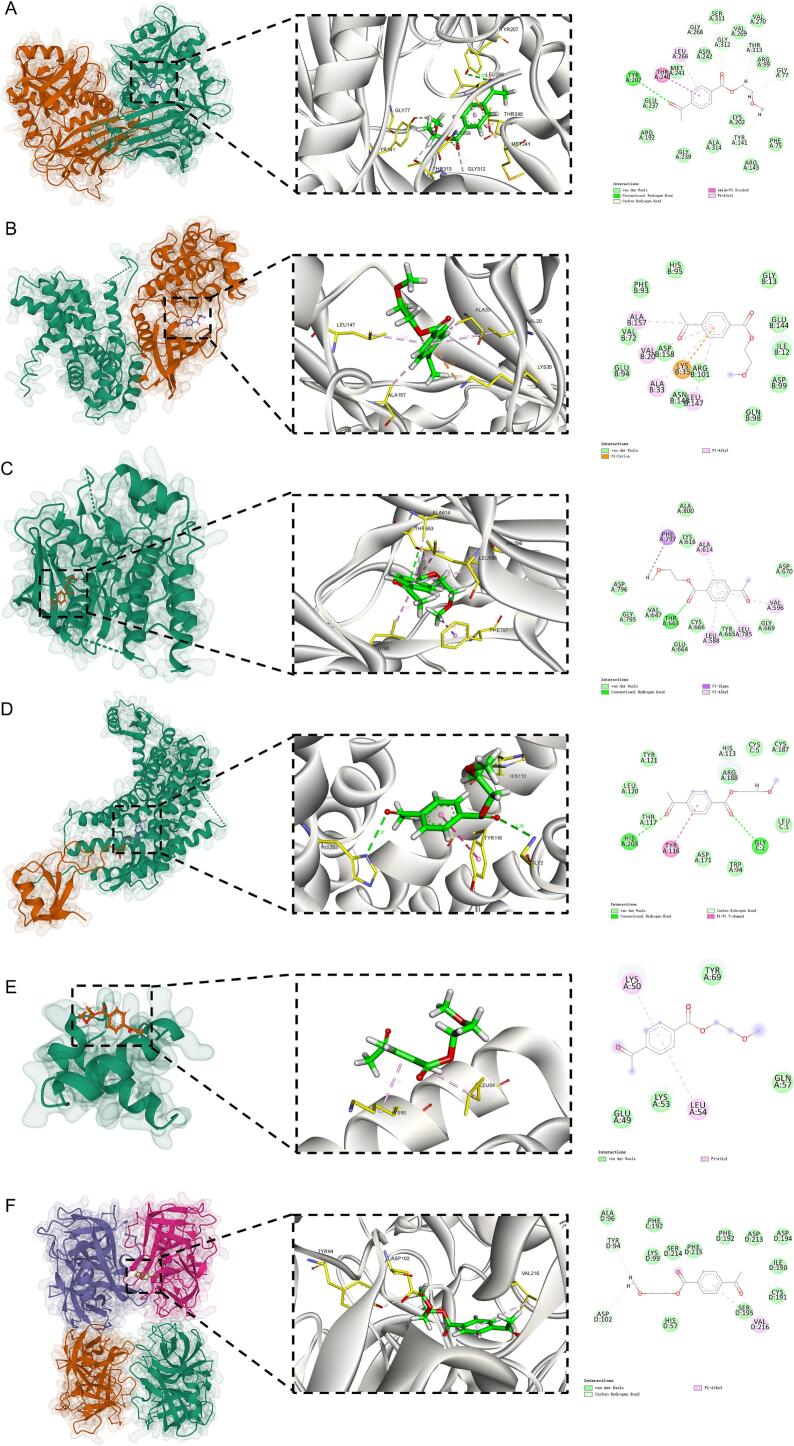
Molecular docking results of PET with six identified hub proteins. (A–F) represent the docking poses of PET with BCAT1 (A), CDK4 (B), CSF1R (C), CXCR4 (D), MYB (E), and PRTN3 (F), respectively. Left: The surface representation of the protein structure with PET docked in the predicted active pocket; Middle: A close-up of the binding site showing the interaction details between PET and surrounding amino acid residues; Right: 2D interaction diagram illustrating various molecular forces between PET and residues at the binding site.

We performed molecular docking simulations between PET and six hub genes to evaluate their potential interactions. The results showed that PET could form stable interactions with the surface active sites of most target proteins, with binding energies below − 5 kcal/mol. Among them, PET exhibited the strongest binding affinity with CSF1R (−8.312 kcal/mol), suggesting it may represent a key target. The binding energies for the other proteins were as follows: BCAT1, −6.656 kcal/mol; CDK4, −6.107 kcal/mol; CXCR4, −5.214 kcal/mol; MYB, −4.311 kcal/mol; and PRTN3, −5.596 kcal/mol. The corresponding binding conformations are shown in Fig. 6A–F.

### 3.6 Drug prediction

**Fig. 7:**
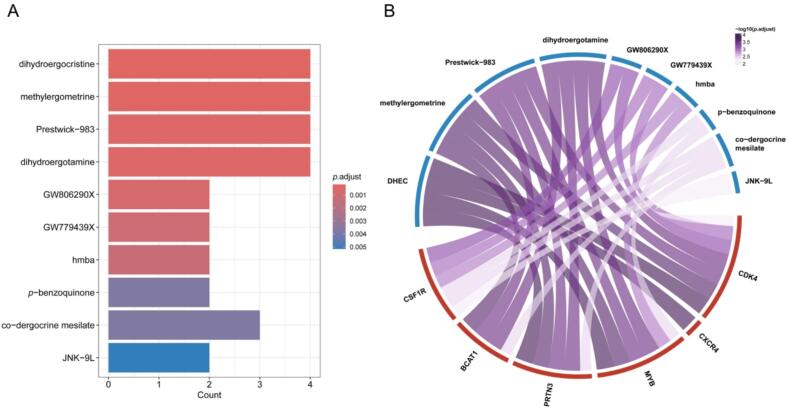
Identification of candidate drugs targeting hub genes. (A) Bar plot showing the top 10 enriched drugs predicted to interact with hub genes. The x-axis indicates the number of overlapped hub genes, and the bar color represents the value of p.adjust. (B) Chord diagram depicting the associations between the top 10 drugs and six hub genes (BCAT1, CDK4, CSF1R, CXCR4, MYB, and PRTN3). The ribbon colors are mapped to − log10(*p*.adjust).

Using the DSigDB database, we identified potential therapeutic compounds targeting the hub genes. Fig. 7 shows the top 10 candidate compounds ranked by adjusted *p*-values. Among them, dihydroergocristine, dihydroergotamine, *p*-Benzoquinone, and JNK-9L have been previously reported to possess antitumor potential in previous studies [[60], [61], [62], [63]]. Notably, *p*-Benzoquinone and JNK-9L may influence OS progression by modulating the PI3K-AKT signaling pathway. Our KEGG enrichment analysis had already identified this pathway as a key PET exposure-related mechanism in OS development. Moreover, PI3K-AKT signaling is widely recognized as a critical regulator of autophagy and has been implicated in various malignancies and pathological conditions [64,65]. These findings provide further evidence supporting the hypothesis that PET exposure may drive OS progression through PI3K-AKT signaling, highlighting *p*-Benzoquinone and JNK-9L as promising therapeutic candidates for mitigating PET-associated OS risks.

## 4 Discussion

Previous studies have confirmed that PET and its degraded microplastic components can enter the human body through multiple exposure pathways, including ingestion (via food), inhalation, and direct skin contact, which can be hazardous to human health [6,7]. With the detection of microplastic components in placenta and blood, it raises concerns about children's health, especially because of the high incidence of OS in children and adolescents [66]. However, whether long-term PET exposure contributes to the occurrence of OS remains unclear. Therefore, we employed a multi-source machine learning integration approach, incorporating data from multiple sources, to systematically analyze the molecular mechanisms underlying OS induction by prolonged PET exposure. We successfully identified key target genes associated with PET-induced OS and elucidated their potential oncogenic mechanisms. These findings provide some theoretical support for future environmental protection and disease prevention.

We identified 12 OS-related genes that may be associated with PET exposure through network toxicology and bioinformatics tools, and GO analysis showed that these intersecting genes may affect OS progression through multiple key biological functions. Among them, ROS plays a crucial role in regulating the cell cycle, inflammation, migration, invasion, and apoptosis, and when overexpressed may induce DNA damage and promote OS progression through oxidative stress [67]. Dysregulation of protein tyrosine kinase activity and protein kinase regulator activity accelerates cell cycle progression while inhibiting apoptotic pathways, ultimately fostering tumor cell proliferation and survival [48]. Additionally, cytokine binding suggests a potential role of the inflammatory microenvironment in OS development [68]. KEGG analysis indicated that the PI3K-Akt signaling pathway enhances OS cell proliferation and drug resistance [69], while the T-cell receptor and chemokine signaling pathways imply that PET exposure may contribute to immune evasion by altering the tumor immune microenvironment [70]. Furthermore, the osteoclast differentiation pathway suggests that PET may promote OS by destroying bone microstructure [71].

To enhance the accuracy of hub genes prediction, we constructed machine learning models and identified glmBoost + LDA as the optimal model. Based on this model, we identified BCAT1, CDK4, CSF1R, CXCR4, MYB, and PRTN3 as hub genes associated with PET exposure-induced OS. Notably, these six hub genes exhibited high AUC values, indicating their significant diagnostic potential and clinical applicability. Furthermore, we observed a strong positive correlation between PRTN3 and CSF1R, suggesting their potential synergistic role in bone microenvironment regulation and immune cell infiltration. CSF1R plays a crucial role in macrophage recruitment and differentiation, while PRTN3, as a serine protease, is involved in inflammatory responses. Their synergistic interaction may enhance tumor-associated immune cell infiltration, thereby promoting OS cell survival, invasion, and immune evasion, ultimately driving tumor progression [72,73]. Additionally, we found that BCAT1 is mainly involved in branched-chain amino acid (BCAA) metabolism and plays a crucial role in tumor growth, proliferation, and apoptosis [74]. Studies have shown that abnormal BCAA metabolism may play a role in cancer through other potential pathways such as the mTOR pathway, glucose and lipid metabolism [75]. BCAT1 may regulate OS cell proliferation by modulating BCAA metabolism [76]. Our study also revealed a strong negative correlation between BCAT1 and PRTN3, which may reflect their opposing roles in metabolic or inflammatory regulation. This mutual antagonistic or compensatory interaction suggests a potential metabolism-inflammation interactions, warranting further investigation to explore its potential value in OS diagnosis and therapy.

To explore the potential mechanisms of hub genes, we conducted KEGG enrichment analysis. We found that these hub genes may play a central role in the PET exposure-induced OS process through the PI3K-Akt signaling pathway. The PI3K/AKT pathway plays a critical role in human life activities, and previous studies have shown that dysregulation of the PI3K/AKT pathway is involved in OS metastasis, cell cycle regulation, apoptosis, angiogenesis, and other pathological processes. Its abnormal activation or dysregulation may be a key factor in OS development [77]. According to our findings, CDK4, CSF1R, and MYB were successfully enriched in the PI3K/AKT pathway, with CSF1R showing the strongest binding to PET (binding energy: −8.312 kcal/mol). Therefore, we speculate that PET may tightly bind to Colony Stimulating Factor 1 Receptor (CSF1R), a tyrosine kinase transmembrane receptor, inducing conformational changes in CSF1R, promoting receptor dimerization and autophosphorylation, and subsequently recruiting the PI3K complex (p85/p110) [78]. PI3K converts PIP2 to PIP3 at the cell membrane, resulting in an increase in the local concentration of PIP3 at the cell membrane. PIP3, as a signaling lipid, recruits PDK1 and AKT to the membrane via its interaction with the PH domain, ultimately activating Akt [79]. Activated Akt enhances the stability of Cyclin D1, which binds to CDK4 to form the Cyclin D/CDK4 complex, driving the G1/S transition in the cell cycle. This results in cell cycle dysregulation and excessive proliferation of OS cells [80,81]. Akt can also promote the expression and activity of MYB, either directly or indirectly. MYB upregulates the expression of Cyclin D1 and CDK4, further driving the cell cycle process [82]. MYB also upregulates anti-apoptotic genes (e.g., Bcl-2) and pro-invasive genes (e.g., MMP9), enhancing the survival and invasiveness of OS cells [83,84].

Further immune cell infiltration analysis suggests that PET exposure may shape the OS immune microenvironment by influencing immune cell recruitment and activation. For example, BCAT1 and CDK4 may play roles in promoting B cell maturation and T cell immune responses, whereas CSF1R and CXCR4 primarily regulate M2 macrophage polarization and tumor immune evasion. Specifically, CSF1R may suppress the activation of certain innate and adaptive immune cells, while CXCR4 might enhance CD8 + T cell function, contributing to immune regulation. Additionally, MYB and PRTN3 are crucial in modulating the functions of B cells, NK cells, and dendritic cells. MYB may facilitate early B cell differentiation, whereas PRTN3 could negatively regulate NK cell quiescence and T cell modulation. These findings indicate that PET exposure may promote OS progression through the intricate regulation of the immune microenvironment, highlighting potential targets for immunotherapy.

Finally, through the DSigDB database, we identified potential therapeutic compounds, among which *p*-Benzoquinone and JNK-9L emerged as key candidates targeting the PI3K-Akt signaling pathway with significant anti-OS potential. *p*-Benzoquinone, an oxidizing agent, has been shown to induce oxidative stress and apoptosis in tumor cells and may inhibit OS cell survival and proliferation by suppressing the PI3K-Akt signaling pathway [62,85]. Additionally, JNK-9L, as an inhibitor of the c-Jun N-terminal kinase (JNK) signaling pathway, may influence cell autophagy and apoptosis through cross-regulation of the PI3K-Akt pathway, thereby inhibiting OS progression [86,87]. Previous studies have demonstrated the important role of the PI3K-Akt signaling pathway in OS development, and our results also showed that the PI3K-Akt signaling pathway was associated with PET exposure, which further supports the feasibility of *p*-Benzoquinone and JNK-9L as potential OS therapeutic agents and provides new insights for precision treatment strategies.

Although our study predicts the mechanisms by which long-term PET exposure may lead to OS from multiple dimensions, including disease targets, signaling pathways, and the immune microenvironment, it lacks direct experimental validation. Computational predictions alone cannot fully capture the complexity of biological systems. Additionally, we did not subdivide the population samples, which may lead to gender, genetic factors, etc. influencing the study results. To further investigate and elucidate the health risks of PET exposure, future research should include large-scale epidemiological studies, particularly in regions with high PET production and among populations with prolonged exposure to plastic products. These studies will provide a solid theoretical foundation for developing prevention and treatment strategies for PET-induced OS and other plastic-related health risks.

## 5 Conclusion

This study systematically analyzes the potential mechanisms through which long-term PET exposure may induce OS via specific molecular pathways by integrating network toxicology, machine learning, and molecular docking techniques. Our research not only provides insights into the potential impact of PET exposure on bone tumors but also plays an important role in future environmental policy-making and implementation. In the future, we will use this study as a basis for further systematic and comprehensive studies to analyze the potential harm of common environmental pollutants to the human skeletal system and explore possible preventive measures. We hope that this study will raise awareness across society regarding the importance of environmental protection, particularly the potential threats of plastic pollution to human health. We aim to encourage relevant authorities to strengthen regulatory oversight and support legislative efforts to reduce the negative impacts of environmental pollution on both human health and ecosystems. Ultimately, our goal is to promote the achievement of green, circular, and sustainable development, contributing to building a healthier ecological environment and the promotion of human health.

## CRediT authorship contribution statement

**Yu Qiao:** Writing – review & editing, Writing – original draft, Visualization, Software, Resources, Methodology, Investigation, Data curation, Conceptualization. **Fahu Yuan:** Writing – review & editing, Validation, Software, Resources, Methodology, Data curation. **Anna Curto-Vilalta:** Writing – review & editing, Methodology. **Rüdiger von Eisenhart-Rothe:** Project administration, Funding acquisition, Supervision, Writing – review and editing. **Florian Hinterwimmer:** Writing – review & editing, Supervision, Project administration, Funding acquisition, Conceptualization.

## Ethics approval and consent to participate

Not applicable.

## Funding

This work was supported by the Nemetschek Innovationsfond and the Bavarian Ministry of Arts and Science.

## Declaration of competing interest

The authors declare that they have no known competing financial interests or personal relationships that could have appeared to influence the work reported in this paper.
