# Supplementary material for: Assessing the mechanism of osteosarcoma induced by long-term PET exposure: prediction from combined network toxicology, machine learning and molecular docking
Source: J Bone Oncol. 2025 Sep 22;55:100714. doi: 10.1016/j.jbo.2025.100714 (PMC12505009; doi:10.1016/j.jbo.2025.100714)
Supplement: Supplementary Data 2 [file mmc2.docx]

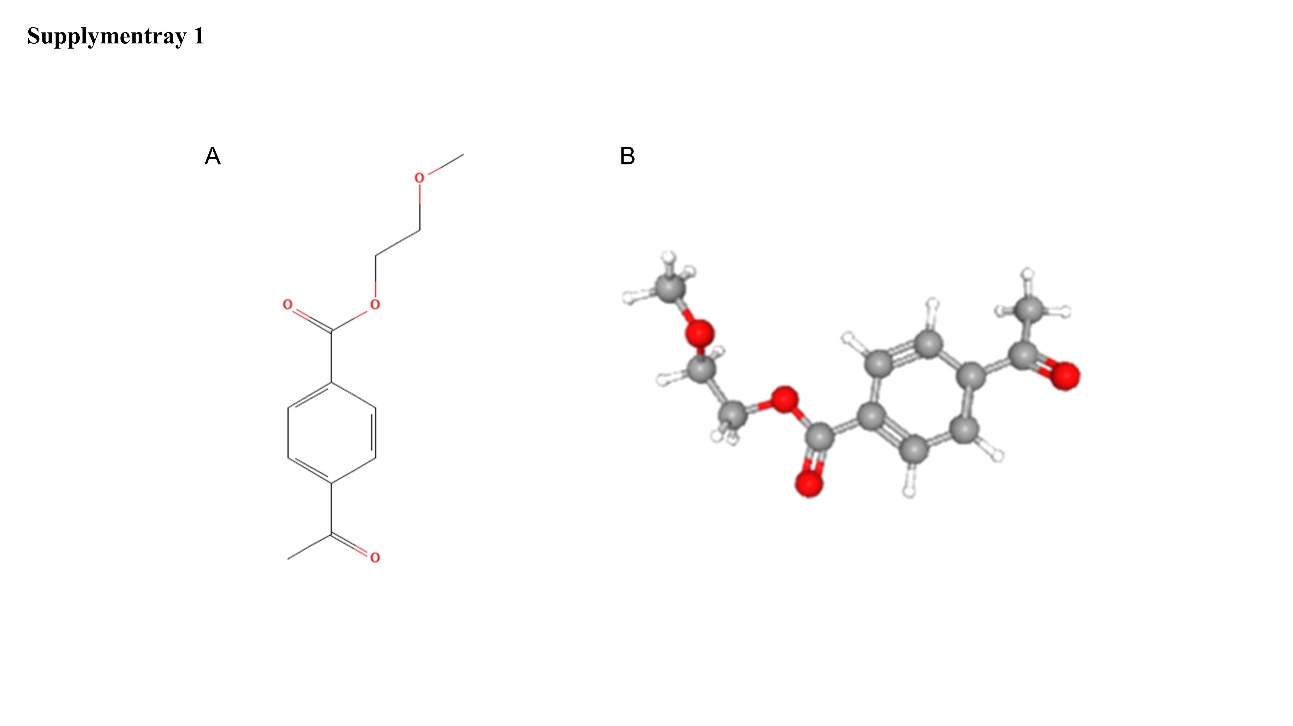


**Supplymentray Figure S1.** Polyethylene terephthalate 2D and 3D structures.


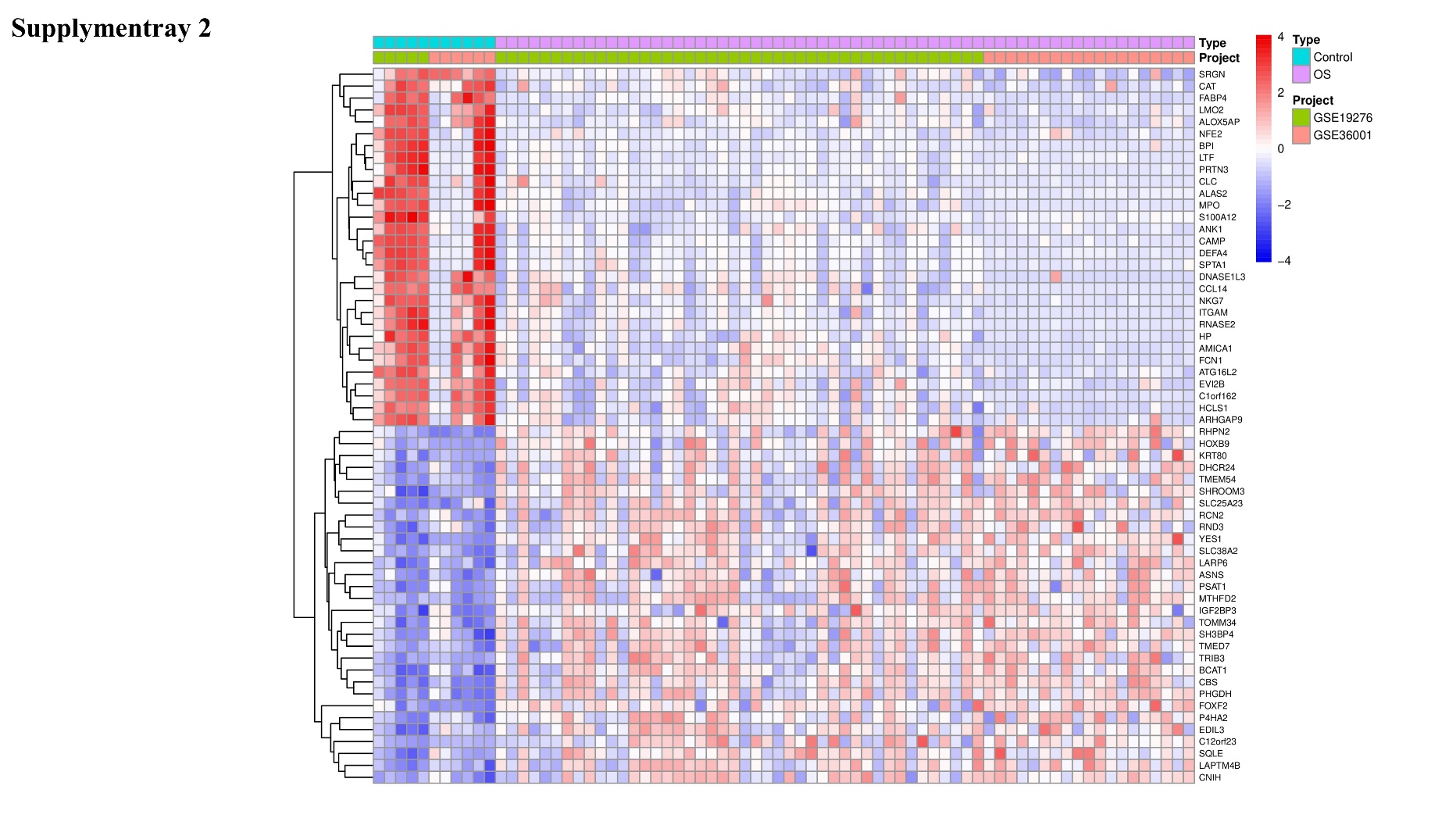


**Supplymentray Figure S2.** Heat map of differentially expressed genes in osteosarcoma.


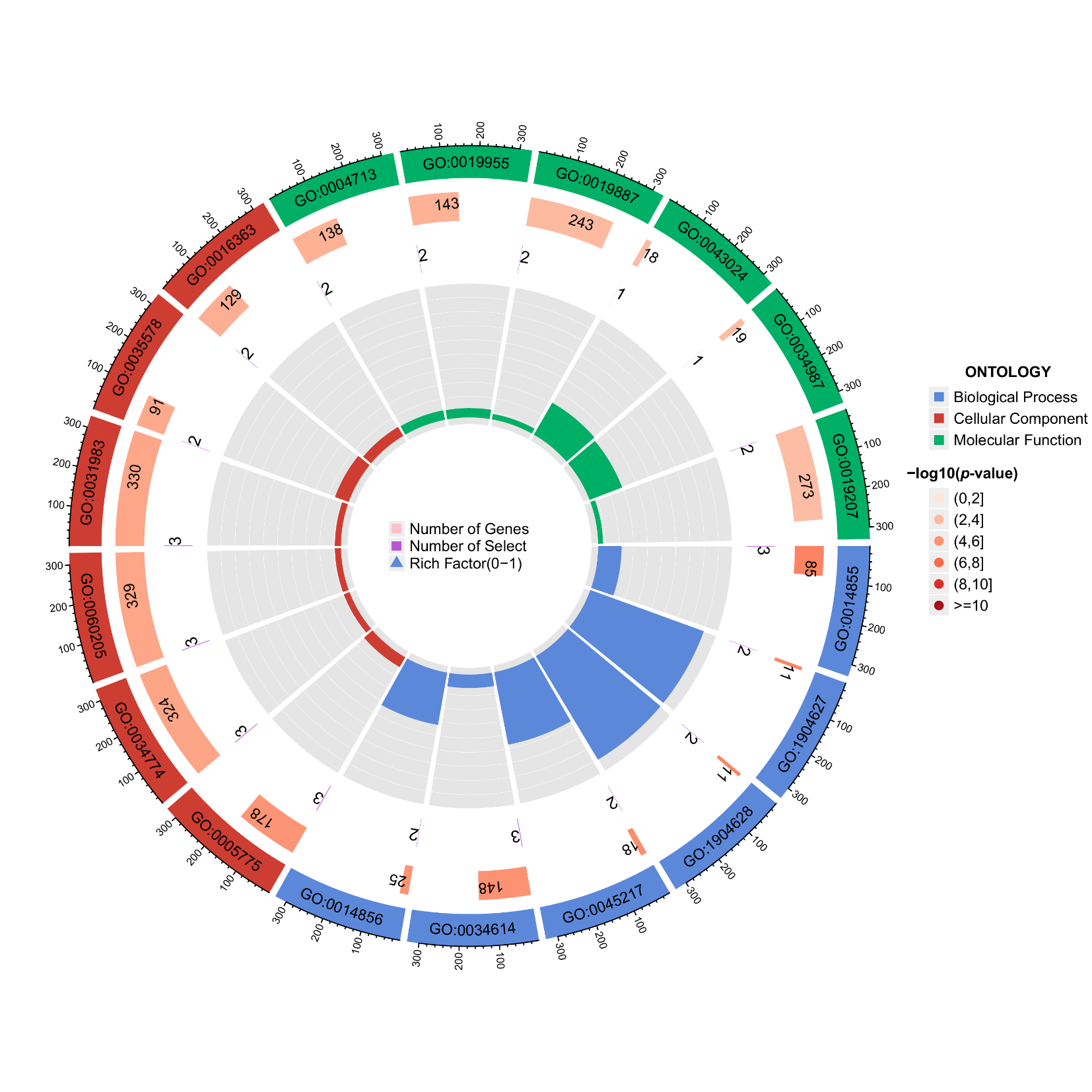


**Supplymentray Figure S3.** Circos plot visualizing GO enrichment analysis of intersecting genes. The outermost circles of blue, red, and green represent biological process (BP), cellular component (CC), and molecular function (MF), respectively, and the inner columns represent the percentage of intersecting genes in the total number of genes in the term.

**Supplymentray Figure S4.** Confusion matrix of the glmBoost+LDA model for the training set and test set. (A) Training set. (B) Training set.


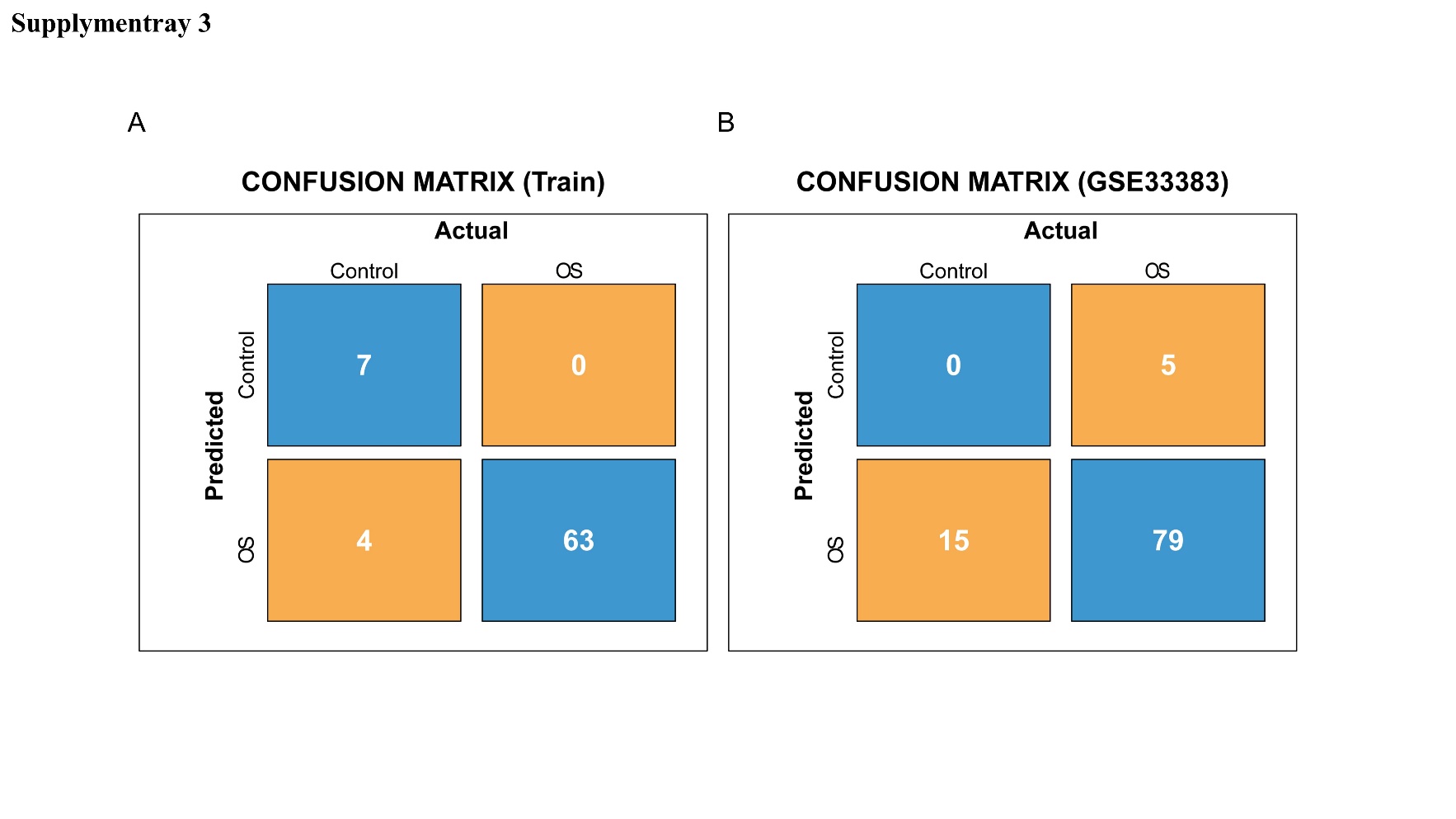


**
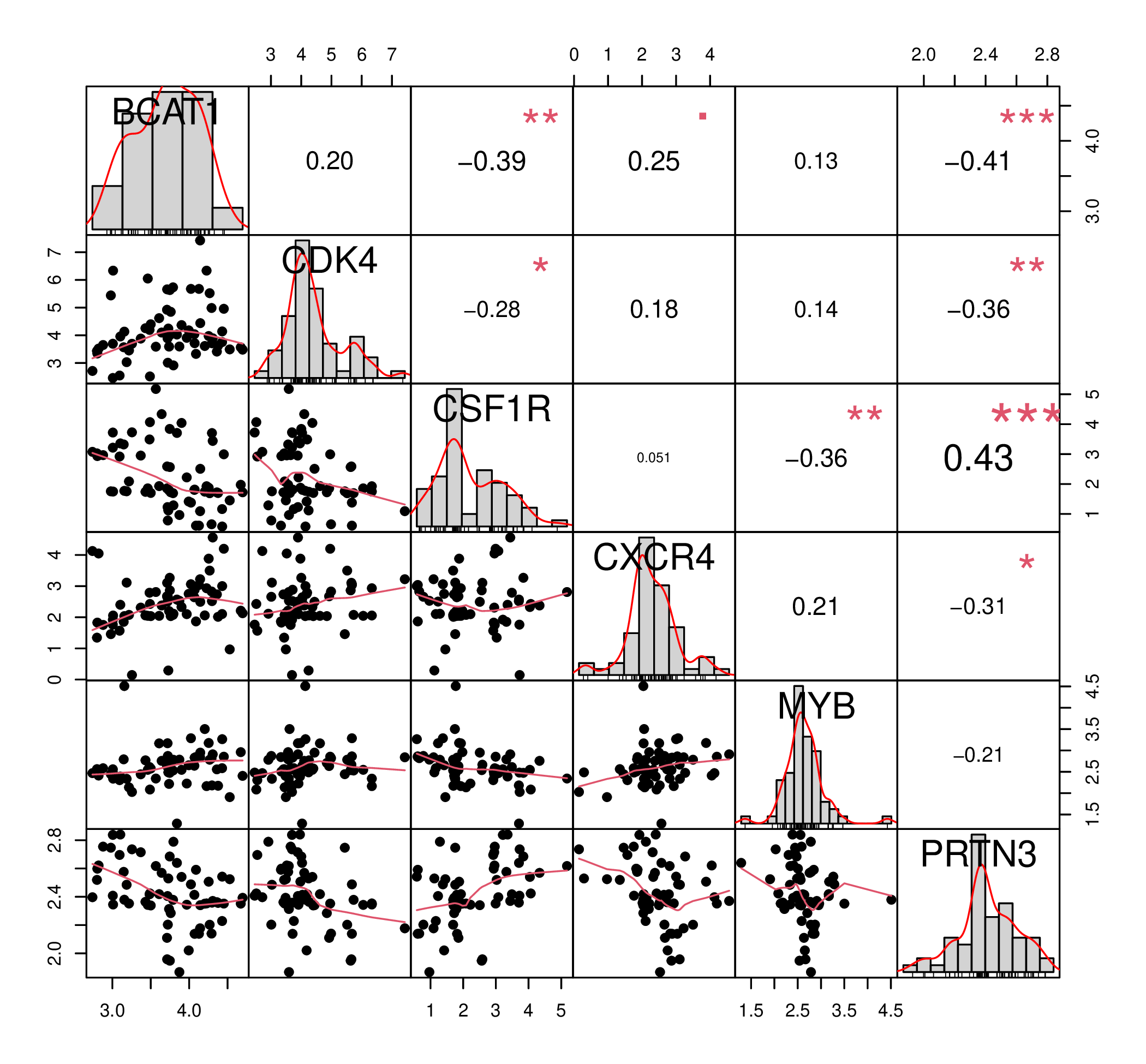
**

**Supplymentray Figure S5.** Correlation matrix illustrating pairwise relationships between hub genes, with significance levels indicated (* p < 0.05, ** p < 0.01, *** p < 0.001).


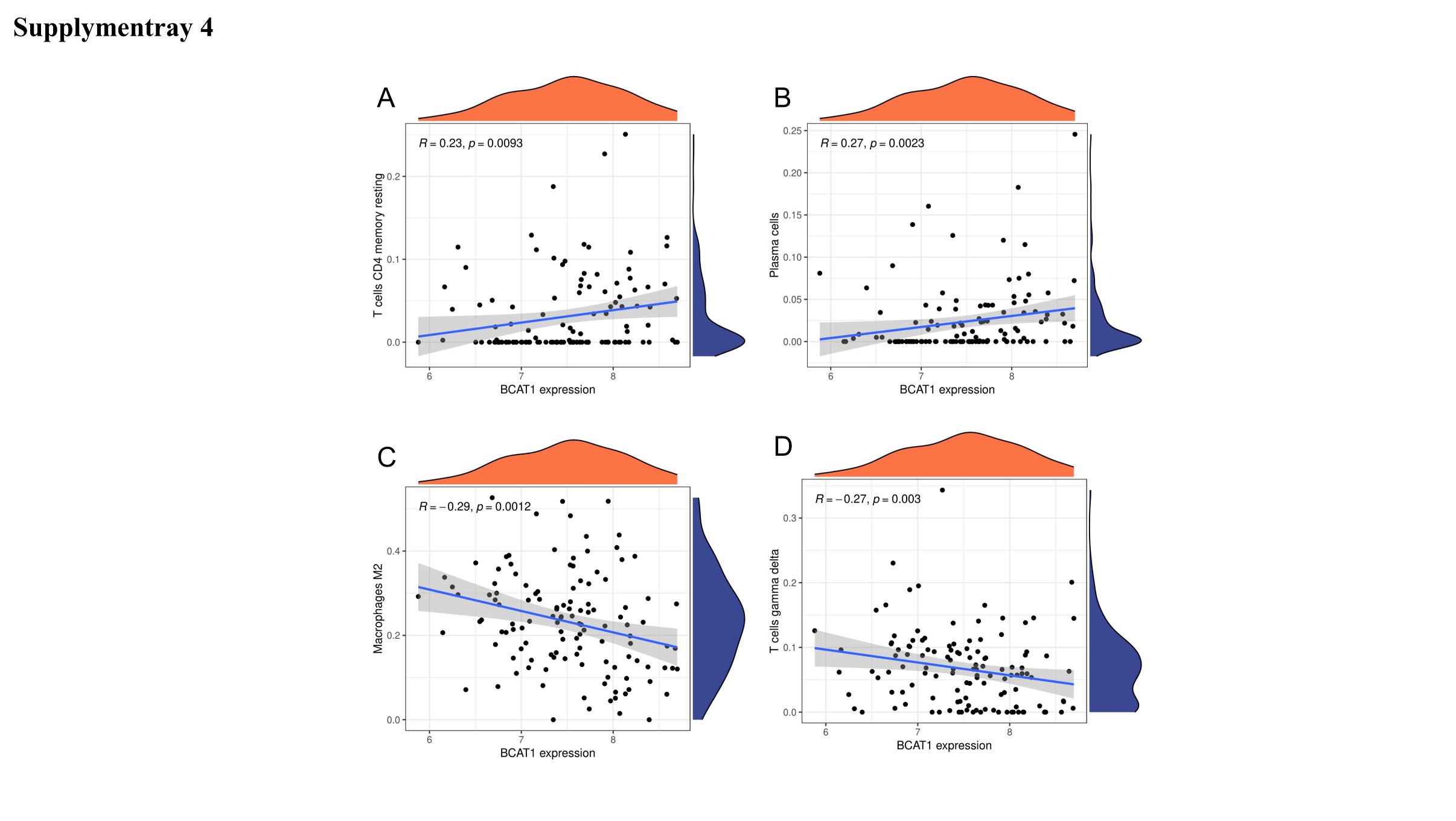


**Supplymentray Figure S6.** Scatter plot of correlation between BCAT1 expression and various immune cell infiltrations. (A) BCAT1 with T cells CD4 memory resting. (B) BCAT1 with Plasma cells. (C) BCAT1 with Macrophages M2. (D) BCAT1 with T cells gamma delta.


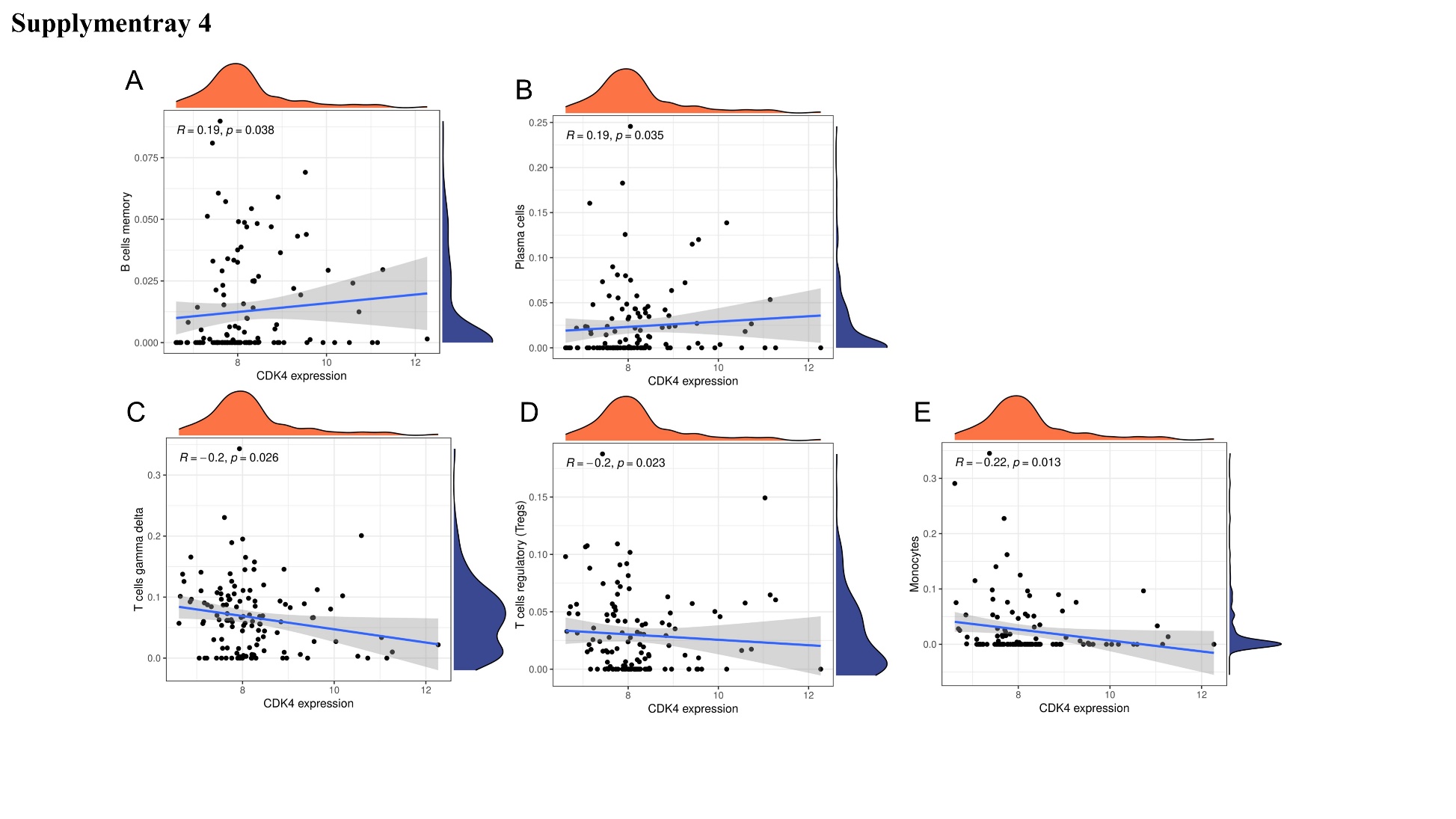


**Supplymentray Figure S7.** Scatter plot of correlation between CDK4 expression and various immune cell infiltrations. (A) CDK4 with B cells memory. (B) CDK4 with Plasma cells. (C) CDK4 with T cells gamma delta. (D) CDK4 with T cells regulatory (Tregs). (E) CDK4 with Monocytes.


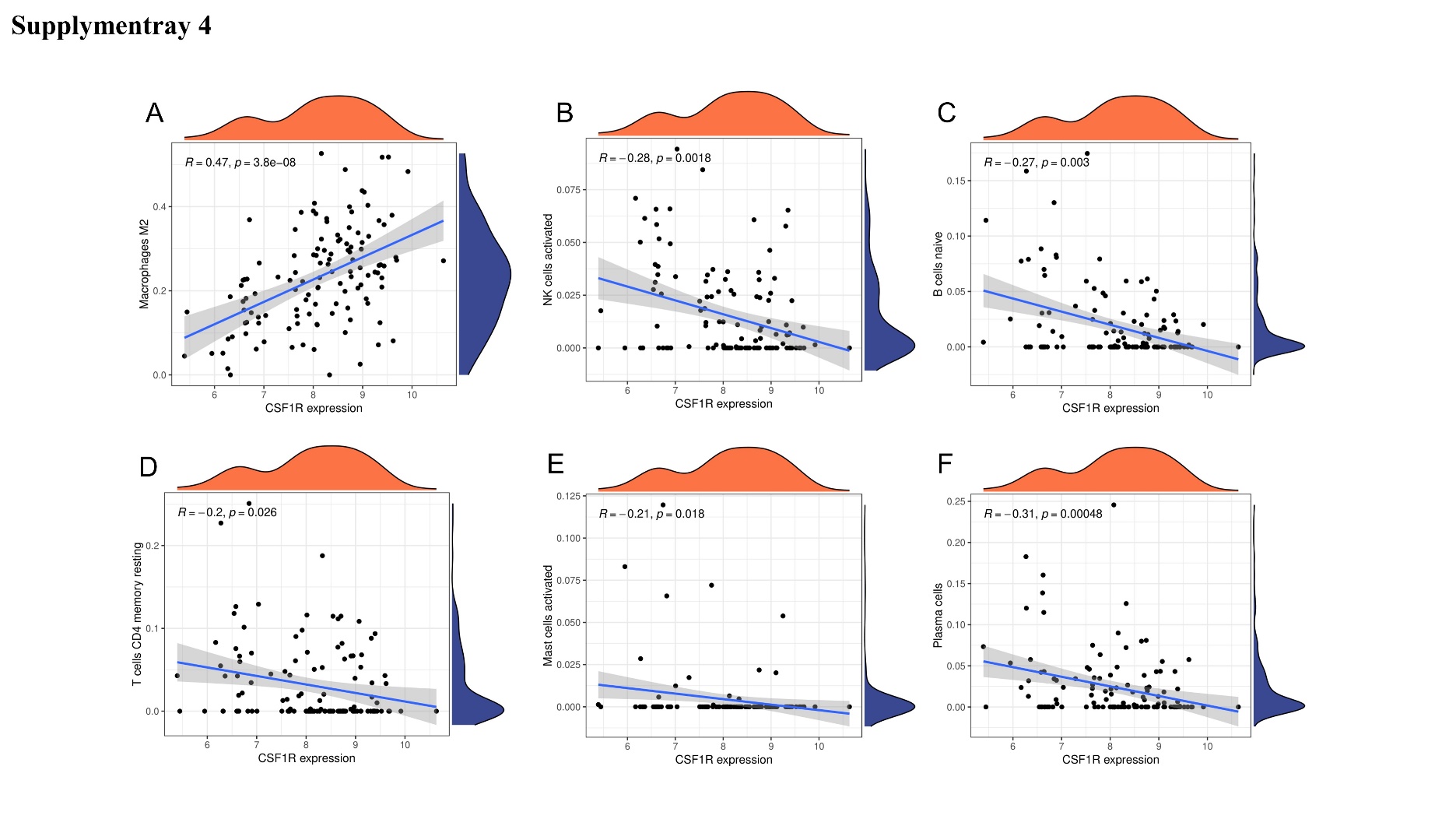


**Supplymentray Figure S8.** Scatter plot of correlation between CSF1R expression and various immune cell infiltrations. (A) CSF1R with Macrophages M2. (B) CSF1R with NK cells activated. (C) CSF1R with B cells naïve. (D) CSF1R with T cells CD4 memory resting. (E) CSF1R with Mast cells activated. (F) CSF1R with Plasma cells.


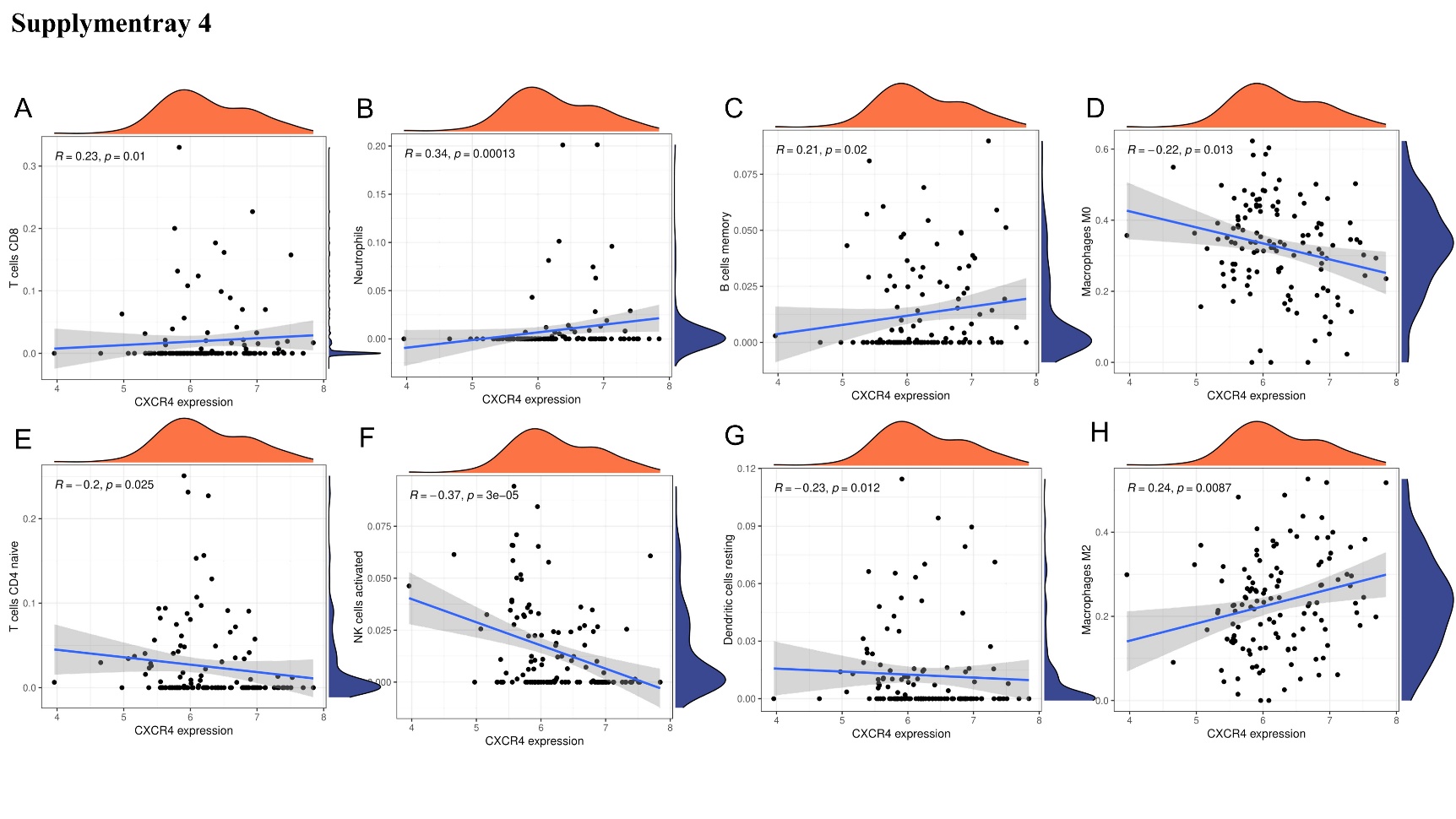


**Supplymentray Figure S9.** Scatter plot of correlation between CXCR4 expression and various immune cell infiltrations. (A) CXCR4 with T cells CD8. (B) CXCR4 with Neutrophils. (C) CXCR4 with B cells memory. (D) CXCR4 with Macrophages M0. (E) CXCR4 with T cells CD4 naive. (F) CXCR4 with NK cells activated. (G) CXCR4 with Dendritic cells resting. (H) CXCR4 with Macrophages M2.


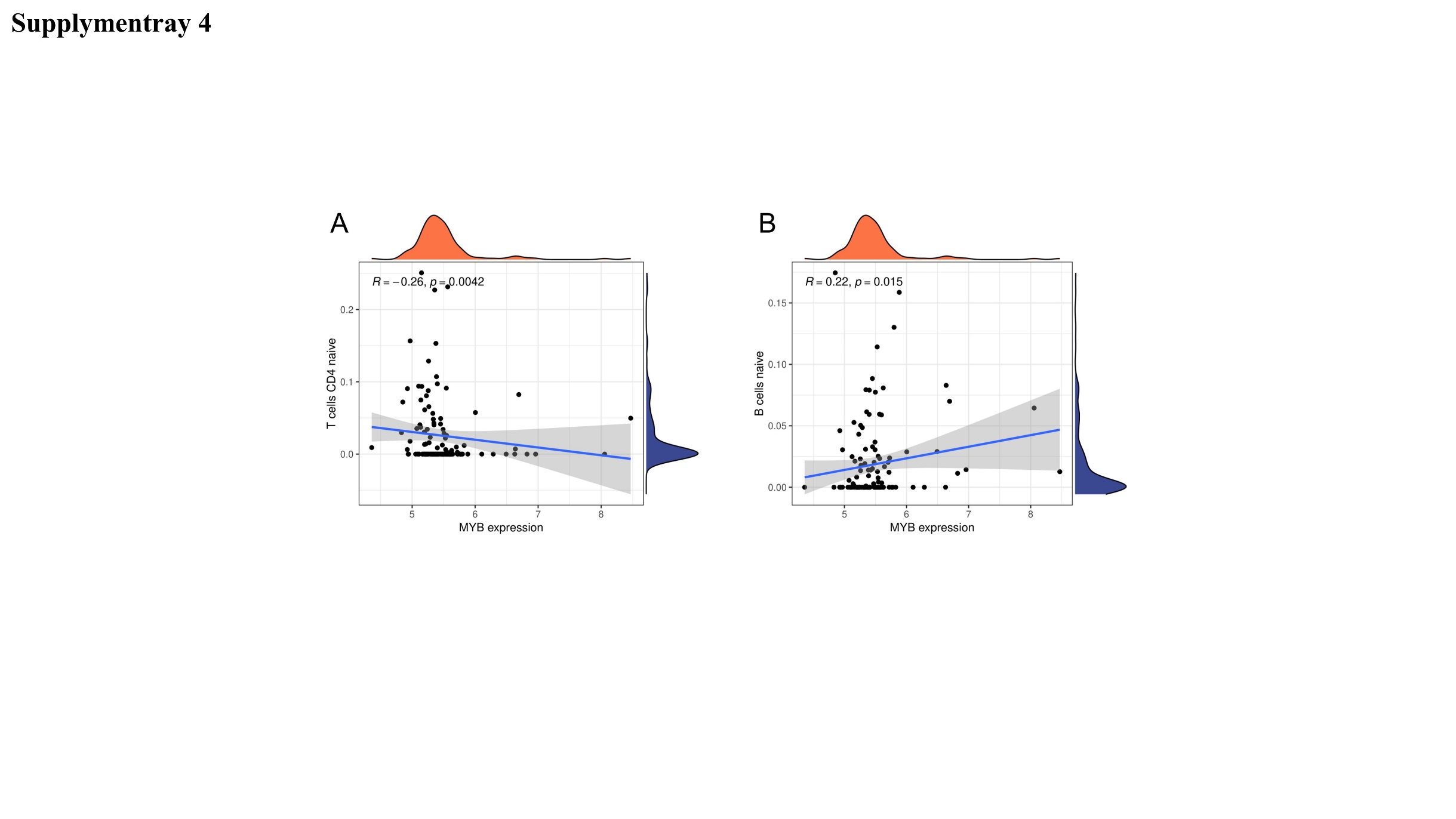


**Supplymentray Figure S10.** Scatter plot of correlation between MYB expression and various immune cell infiltrations. (A) MYB with T cells CD4 naive. (B) MYB with B cells naive.


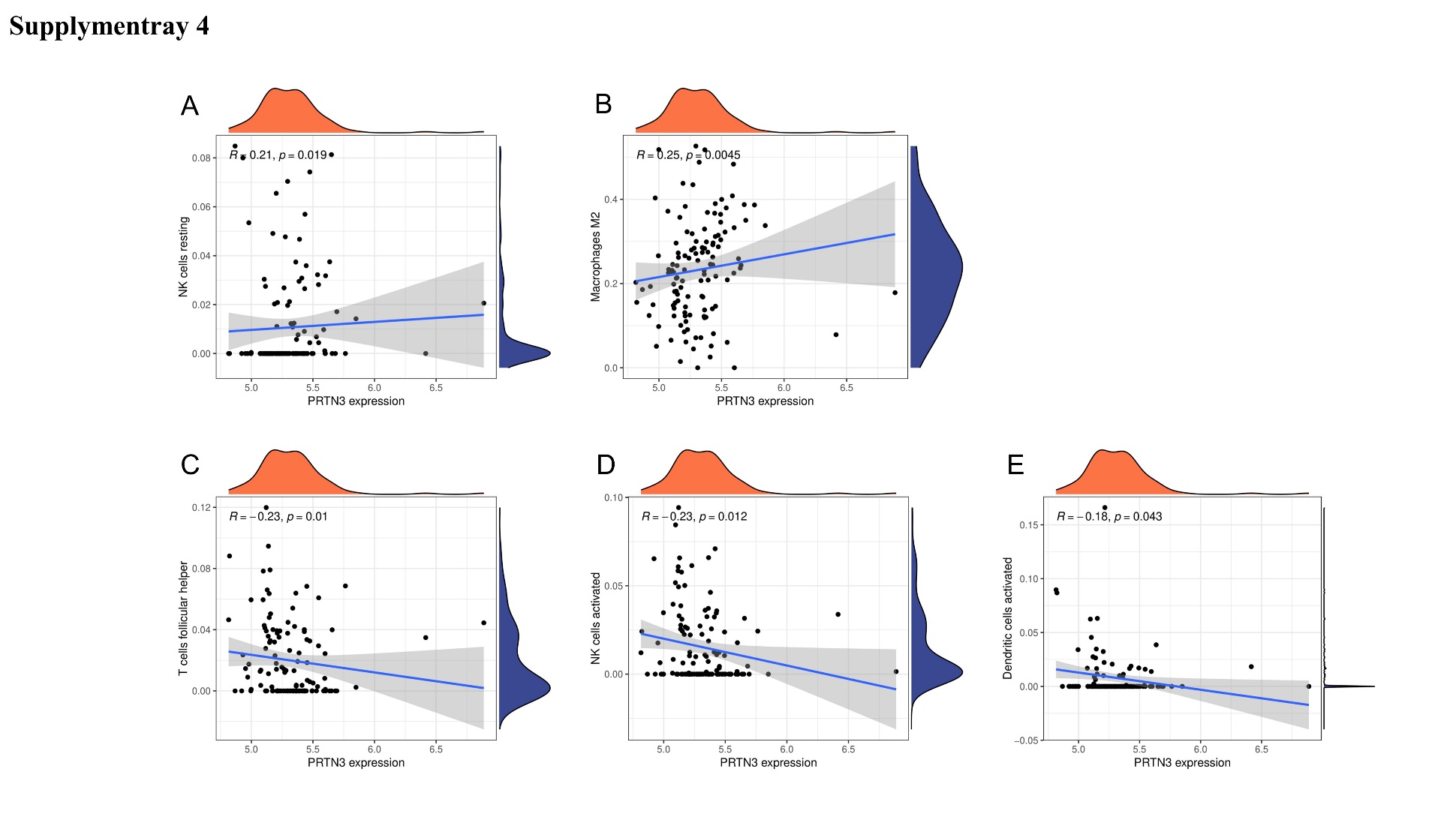


**Supplymentray Figure S11.** Scatter plot of correlation between PRTN3 expression and various immune cell infiltrations. (A) PRTN3 with NK cells resting. (B) PRTN3 with Macrophages M2. (C) PRTN3 with T cells follicular helper. (D) PRTN3 with NK cells activated. (E) PRTN3 with Dendritic cells activated.
